# Regulating Humidity-Dependent Catalyst-Layer Transport by an Ultrathin Carbon Interface for Low-RH PEMFCs

**DOI:** 10.3390/nano16201297

**Published:** 2026-10-13

**Authors:** Caihan Zhu, Dewei Li, Tao Wu, Yunan Wang

**Affiliations:** 1 State Key Laboratory of Advanced Marine Materials, Zhejiang Key Laboratory of Advanced Fuel Cells and Electrolyzers Technology, Ningbo Institute of Materials Technology and Engineering, Chinese Academy of Sciences, Ningbo 315201, China; zhucaihan@nimte.ac.cn (C.Z.); lidewei@nimte.ac.cn (D.L.); 2 Department of Chemical and Environmental Engineering, University of Nottingham Ningbo China, Ningbo 315100, China

**Keywords:** proton exchange membrane fuel cell, nitrogen-doped carbon coating, low humidity, catalyst-layer transport

## Abstract

Proton exchange membrane fuel cells (PEMFCs) suffer substantial performance losses under low relative humidity (RH) because catalyst-layer dehydration compromises proton conduction and interfacial reactant transport. Here, commercial Pt/C was modified through polydopamine deposition followed by thermal treatment to form an ultrathin nitrogen-doped carbon coating (NCS-Pt/C), aiming to regulate hydration and the transport environment within the catalyst layer. The modification largely preserved the porous architecture of the parent Pt/C while increasing water vapor uptake and improving surface wettability. When incorporated into membrane electrode assemblies at a cathode Pt loading of 0.10 mg_Pt_ cm^−2^, NCS-Pt/C delivered higher performance across 30–90% RH. The peak power density increased from 0.35 to 0.50 W cm^−2^ at 30% RH, from 0.57 to 0.67 W cm^−2^ at 60% RH, and from 0.67 to 0.77 W cm^−2^ at 90% RH. Under low-RH conditions, NCS-Pt/C exhibited improved dry proton accessibility and lower apparent catalyst-layer proton-conduction resistance. Substantially lower catalyst-layer oxygen transport resistance was also observed at 90% RH. These results show that the carbon interfacial modification is associated with reduced humidity sensitivity, accompanied by coupled changes in catalyst-layer hydration, interfacial transport, and reaction kinetics, with the benefit becoming particularly pronounced under dry operating conditions.

## 1. Introduction

Proton exchange membrane fuel cells (PEMFCs) require sufficient hydration to sustain efficient proton transport through both the membrane and catalyst layers (CLs) [1,2,3]. For applications such as high-altitude power systems, arid-region operation, and portable devices, maintaining high performance under reduced relative humidity (RH) is desirable because it can lower external humidification requirements and simplify the balance of plant design [4,5,6,7,8]. However, decreasing RH causes progressive dehydration of the CL, which alters the ionomer state and simultaneously affects proton conduction, catalyst utilization, and reactant transport [4,9,10,11]. Specifically, reduced water content forces proton conduction to rely more heavily on ionomer-network connectivity, increasing proton transport tortuosity [12,13,14]. In addition, ionomer contraction under low RH also results in dense coverage around Pt nanoparticles and raises oxygen transport resistance. These effects become increasingly important at low Pt loading, where losses in accessible active sites and local transport efficiency have a greater impact on overall cell performance [15,16,17,18,19]. Therefore, improving catalyst-layer hydration and interfacial transport under reduced humidification is essential for achieving efficient PEMFC operation at low Pt loading [20,21].

Several strategies have been explored to mitigate PEMFC performance losses under reduced humidification by regulating water retention and transport at different levels of the cell. Hydrophilic additives, including γ-Al_2_O_3_, SiO_2_, and polymers containing polar groups, have been incorporated directly into catalyst layers (CLs) to increase local water uptake and help sustain ionomer hydration under low-RH conditions [9,22,23,24,25,26]. In particular, SiO_2_-containing CLs have been shown to increase local water content, reduce ohmic losses, and preserve catalyst utilization under dry operation, leading to substantial improvements in low-RH cell performance [27,28]. However, the effectiveness of such hydrophilic additives depends on their loading and hydration state, as excessive additive content or water accumulation may introduce additional transport penalties. Related inorganic–organic composite membranes have also been developed to sustain proton conduction under low humidity conditions through ionomer–oxide interactions and improved utilization or retention of interfacial water [29,30]. Water distribution can also be regulated through hydrophilic interlayers in gas diffusion media or through structured electrodes, such as electrospun nanofiber CLs and micropatterned interfaces [31]. While these strategies can improve low-RH performance, they generally rely on additional functional phases, modification of other cell components, or more complex electrode architectures. This motivates catalyst-level interfacial approaches that can regulate hydration and transport within conventional CL structures without substantially altering established electrode fabrication processes.

Surface modification provides a practical route to tune catalyst–ionomer interactions without substantially changing conventional electrode fabrication processes [32,33,34]. Orfanidi et al. functionalized carbon supports with amino-containing surface groups and demonstrated altered interactions with sulfonated ionomers [35]. Ott et al. further showed that nitrogen-containing carbon functionalities, including pyridinic, pyrrolic, and graphitic N species, can modify catalyst–ionomer interactions and affect the resulting ionomer distribution [33]. These studies motivate control of surface chemistry, but the resulting effects depend on catalyst composition, ink formulation, and operating conditions.

Carbon shell modification has emerged as a useful approach for tailoring the interfacial properties of metal catalysts while maintaining the structural framework of conventional supported catalysts. In particular, ultrathin N-doped carbon coatings have been reported to modify the electronic environment of Pt and mitigate the specific adsorption of ionomers on Pt surfaces [36]. However, previous studies have focused primarily on Pt surface chemistry and ionomer adsorption, whereas the influence of such interfacial modification on catalyst-layer behavior under varying humidity remains less understood. How the modified interface affects water affinity, proton accessibility, and transport characteristics as the catalyst layer becomes dehydrated has not been systematically clarified. Here, commercial Pt/C was modified by polydopamine deposition followed by thermal treatment to form an ultrathin N-doped carbon coating (NCS-Pt/C). The catalyst and the resulting catalyst layers were evaluated over a wide RH range to examine the effects of the coating on water affinity, electrochemical activity, proton transport, oxygen transport, and cell performance at a cathode Pt loading of 0.10 mg_Pt_ cm^−2^. Rather than treating the carbon coating solely as a protective layer for Pt, this work focuses on its influence on the humidity-dependent hydration and transport environment within practical catalyst layers, providing insight into interfacial strategies for improving PEMFC operation under reduced humidification.

## 2. Materials and Methods

### 2.1. Preparation of an N-Doped, Carbon-Modified Pt/C

An N-doped carbon shell was encapsulated on the surface of a commercial Pt/C (hereafter denoted Com-Pt/C) catalyst (TEC10E50E, Tanaka Kikinzoku Kogyo, Tokyo, Japan, 47 wt% Pt) through a self-polymerization process of dopamine followed by carbonization. Briefly, 6 mg of dopamine hydrochloride (98%) was dissolved in 20 mL of Tris–HCl buffer solution (10 mM, pH = 8.5). Subsequently, 40 mg of the Pt/C catalyst was added to the solution, and the mixture was magnetically stirred at 60 °C for 90 min to facilitate the self-polymerization of dopamine and the formation of a polydopamine coating. The resulting powder was collected by centrifugation, washed with deionized water, and dried under vacuum at 30 °C for 24 h. Finally, the powder was subjected to thermal treatment in a tube furnace at 450 °C for 2 h under an N_2_ atmosphere to convert the polydopamine (PDA) coating into an N-doped carbonaceous coating. The resulting catalyst contained 43.8 wt% Pt as determined by TGA; the lower Pt mass fraction relative to the pristine Pt/C reflects the additional mass introduced by the carbonaceous coating rather than a lower Pt loading in the electrode. The catalyst is hereafter referred to as NCS-Pt/C.

### 2.2. Physical Characterization

Transmission electron microscopy (TEM) observations were performed using a JEOL JEM-2100F microscope (JEOL Ltd., Akishima, Japan) operated at 200 kV and equipped with an EDAX Genesis energy-dispersive X-ray spectroscopy (EDS) system (EDAX Inc., Mahwah, NJ, USA). X-ray photoelectron spectroscopy (XPS) measurements were carried out using a Thermo Scientific K-Alpha spectrometer (Thermo Fisher Scientific, East Grinstead, UK) with monochromatic Al Kα radiation (hν = 1486.6 eV). Survey spectra were acquired at a pass energy of 150 eV with a step size of 1.0 eV, while high-resolution spectra were collected at 50 eV with a step size of 0.1 eV. Binding energies were calibrated against the C 1s peak at 284.8 eV, and the spectra were analyzed using Avantage software (version 5.948, Thermo Fisher Scientific, East Grinstead, UK). Thermogravimetric analysis (TGA) was performed using a PerkinElmer TGA 8000-Spectrum Two-Clarus SQ8T system (PerkinElmer, Waltham, MA, USA) under an air atmosphere, with the temperature increased from room temperature to 900 °C at a heating rate of 10 °C min^−1^. Nitrogen adsorption–desorption isotherms were measured at 77 K using a BELSORP MAX analyzer (MicrotracBEL Corp., Osaka, Japan), with specific surface areas and pore size distributions determined by the BET and BJH methods, respectively. Water vapor adsorption isotherms were measured at 25 °C using a Micromeritics ASAP 2020 analyzer (Micromeritics Instrument Corp., Norcross, GA, USA). Zeta potential and particle size distributions were measured using an OMEC NS-Zeta analyzer (Zhuhai OMEC Instruments Co., Ltd., Zhuhai, China) and a Bettersize BT-9300ST analyzer (Bettersize Instruments Ltd., Dandong, China), respectively. Static water contact angles of the catalyst layers were measured using the sessile drop method.

### 2.3. Rotating Disk Electrode Testing

Electrochemical measurements were performed using a Biologic SP-150e potentiostat (BioLogic Science Instruments, Seyssinet-Pariset, France) in a standard three-electrode configuration at 25 °C, with a glassy carbon rotating disk electrode (0.196 cm^2^) as the working electrode, an Ag/AgCl electrode as the reference electrode, and a platinum net as the counter electrode, where all potentials are referenced to the reversible hydrogen electrode (RHE). The catalyst ink was prepared by ultrasonically dispersing the catalyst in a mixture of 2-propanol and deionized water, followed by addition of ionomer solution, and then drop-cast onto the glassy carbon electrode and dried at room temperature to form a uniform film, with Pt loadings of 12.7 μg_Pt_·cm^−2^ for the NCS-Pt/C catalyst and 13.5 μg_Pt_·cm^−2^ for the Com-Pt/C. Cyclic voltammetry (CV) was conducted in N_2_-saturated 0.1 M HClO4 by scanning from 0.05 to 1.2 V vs. RHE at 50 mV·s^−1^, and the electrochemical surface area (ECSA) was calculated from the hydrogen desorption charge using 210 μC·cmPt^−2^ as the conversion factor. The oxygen reduction reaction (ORR) activity was evaluated via linear sweep voltammetry (LSV) at a rotation rate of 1600 rpm, with background scans recorded in an N_2_-saturated electrolyte at 20 mV·s^−1^ prior to ORR polarization measurements in an O_2_-saturated electrolyte.

### 2.4. Preparation of Catalyst Layers and Pt Loading Determination

The catalyst ink for the cathode was prepared by mixing the catalyst (NCS-Pt/C or Com-Pt/C) with ionomer (Aquivion D79-25BS), deionized water (18.2 MΩ·cm), and ethanol, followed by ultrasonication for 30 min. The cathode ink had a solid content of 0.8 wt%, an ionomer-to-carbon (I/C) mass ratio of 0.47, and a water/ethanol mass ratio of 0.25. The anode ink was prepared similarly using a commercial Pt/C catalyst (TEC10E20E, Tanaka Kikinzoku Kogyo, 20 wt% Pt) with a solid content of 1 wt%, an I/C ratio of 0.7, and the same water-to-ethanol ratio.

The CLs were fabricated via ultrasonic spraying (XF-400, Suzhou Xifeng Automation Co., Ltd., Suzhou, China) onto a PEM with an active area of 4 cm^2^. The Pt loadings were determined by X-ray fluorescence spectroscopy (XRF, MESA-50, HORIBA, Ltd., Kyoto, Japan) and were measured to be 0.10 mg_Pt_·cm^−2^ for the cathode and 0.03 mg_Pt_·cm^−2^ for the anode. The XRF measurements were conducted using a tube voltage of 50 kV and a tube current of 4 μA. Five locations were selected for each sample, and each location was tested for 30 s; the final average value was then taken. Hereafter, the CL made by NCS-Pt/C is referred to as NCS-Pt/C CL and the CL made by Com-Pt/C as Com-Pt/C CL.

### 2.5. Single-Cell Electrochemical Characterization

Electrochemical characterization included polarization curves, cyclic voltammetry (CV), potentiostatic electrochemical impedance spectroscopy (PEIS), and limiting current measurements. Polarization curves were recorded at 80 °C with H_2_ and air supplied at 1.0 and 2.0 NL min^−1^, respectively, at an absolute outlet pressure of 170 kPa using a parallel flow field.

Voltage loss analysis was further performed using the measured polarization curves. The reversible cell voltage $E_{rev}$ was calculated according to Equation (1):

(1) $$E_{rev}=1.229-8.46\times 10^{-4}\left(T-298.15\right)+\frac{RT}{nF}\ln\left(P_{H_{2}}P_{O_{2}}^{\frac{1}{2}}\right)$$

where T is the cell temperature, R is the universal gas constant, F is the Faraday constant, n = 2 is the number of electrons transferred in the overall hydrogen oxidation/oxygen reduction reaction, and $P_{H_{2}}\;$and $P_{O_{2}}$ are the hydrogen and oxygen partial pressures, respectively. For thermodynamic consistency, the gas pressures are expressed relative to the standard pressure.

The activation overpotential was determined from the Tafel relationship in the kinetically controlled region:

(2) $$\eta_{act}=a+b{log}_{10}\left(i\right)$$

where i is the current density, and a and b are the fitted Tafel parameters. The ohmic overpotential was calculated from the high-frequency resistance $R_{ohm}$ as

(3) $$\eta_{ohm}=iR_{ohm}$$

and the corresponding IR-free cell voltage was obtained from

(4) $$V_{IR-free}=V+\eta_{ohm}=V+iR_{ohm}$$

where V is the measured cell voltage. The residual transport-related overpotential was then calculated as

(5) $$\eta_{mt}=E_{rev}-V_{IR-free}-\eta_{act}$$

which is equivalently expressed as

(6) $$\eta_{mt}=E_{rev}-V-\eta_{ohm}-\eta_{act}$$

Here, $\eta_{mt}$ is treated as a transport-related voltage loss rather than a purely oxygen mass transport overpotential, because under reduced humidity conditions it may contain contributions from both reactant transport and distributed proton-conduction limitations within the catalyst layer.

For CV measurements, the cell was maintained at 40 °C with H_2_ supplied to the anode at 0.2 NL min^−1^. The cathode was first purged with N_2_ until a stable inert atmosphere was established, after which the N_2_ flow was stopped, and CV was recorded under static N_2_ conditions. The ECSA was determined from the hydrogen-adsorption charge on the cathodic scan by integrating the hydrogen underpotential deposition (H_UPD_) region between 0.10 and 0.40 V after subtraction of the double-layer current. The ECSA was calculated according to Equation (7):

(7) $$ECSA=Q_{H}/{(\Gamma }_{H}m_{Pt})$$

where $Q_{H}$ is the integrated hydrogen-adsorption charge, $m_{Pt}$ is the cathode Pt mass, and $\Gamma_{H}$ is the charge density for a monolayer of adsorbed hydrogen on Pt (210 μC cm_Pt_^−2^).

PEIS measurements were performed at 80 °C under H_2_/N_2_ with both gases supplied at 0.2 NL min^−1^, using a DC potential of 0.4 V and a 20 mV AC perturbation over a frequency range of 100 kHz–0.1 Hz. The apparent catalyst-layer proton-conduction resistance ($R_{H^{+}}$) was estimated from the real axis span of the impedance response under blocking conditions according to Equation (8):

(8) $$R_{H^{+}}=3\left(R_{LF}-R_{HF}\right)=3\Delta R$$

where $R_{HF}$ and $R_{LF}$ are the high- and low-frequency real axis intercepts, respectively. The factor of three arises from the distributed proton-conduction resistance of a homogeneous catalyst layer under blocking conditions.

Limiting current measurements were conducted under H_2_/1% O_2_–99% N_2_ at absolute pressures of 100, 200, and 300 kPa. The potential was first swept from 0.15 to 0.35 V at 5 mV s^−1^ to identify the limiting current region, followed by chronoamperometry at a selected potential within this region until a stable current was obtained. The total oxygen transport resistance ($R_{O_{2}}^{total}$) was calculated from the steady-state limiting current density ($i_{lim}$) according to Equation (9):

(9) $$R_{O_{2}}^{total}=4FC_{O_{2}}/i_{lim}$$

where F is the Faraday constant, and $C_{O_{2}}$ is the oxygen concentration in the cathode gas channel, calculated as Equation (10):

(10) $$C_{O_{2}}=x_{O_{2}}\left(P_{abs}-P_{H_{2}O}\right)/RT$$

where $x_{O_{2}}$ is the dry oxygen mole fraction, $P_{abs}$ is the absolute pressure, $P_{H_{2}O}$ is the water vapor partial pressure, R is the universal gas constant, and T is the cell temperature. The pressure-dependent and pressure-independent contributions were separated by linear regression of $R_{O_{2}}^{total}$ against absolute pressure:

(11) $$R_{O_{2}}^{total}=kP_{abs}+R_{O_{2}}^{P-ind}$$

where $kP_{abs}$ represents the pressure-dependent contribution, and the intercept $R_{O_{2}}^{P-ind}$ represents the pressure-independent oxygen transport resistance. In this work, the latter is used as the apparent catalyst-layer-associated oxygen transport resistance (R_CL_).

## 3. Results

### 3.1. Physicochemical Properties of Com-Pt/C and NCS-Pt/C

**Figure 1:**
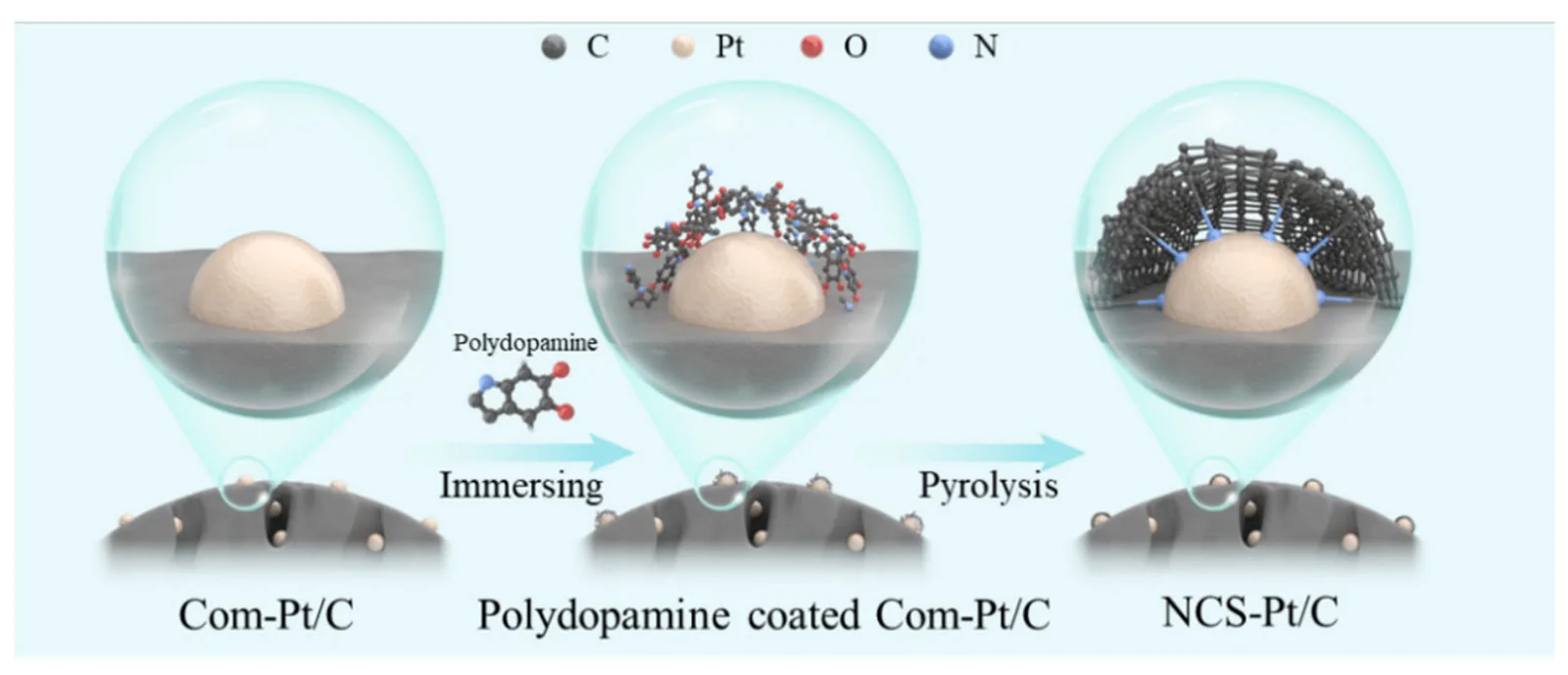
Schematic illustration of the preparation of an N-doped, carbon-modified Pt/C (NCS-Pt/C).

An N-doped, carbon-modified Pt/C catalyst (NCS-Pt/C) was prepared from commercial Pt/C (TEC10E50E) through dopamine self-polymerization followed by thermal carbonization, as illustrated in Figure 1. Dopamine was first polymerized in an alkaline Tris–HCl buffer (pH 8.5), forming a polydopamine (PDA) layer on the catalyst surface. Subsequent heat treatment under N_2_ converted the PDA precursor into a thin N-containing carbonaceous coating.

**Figure 2:**
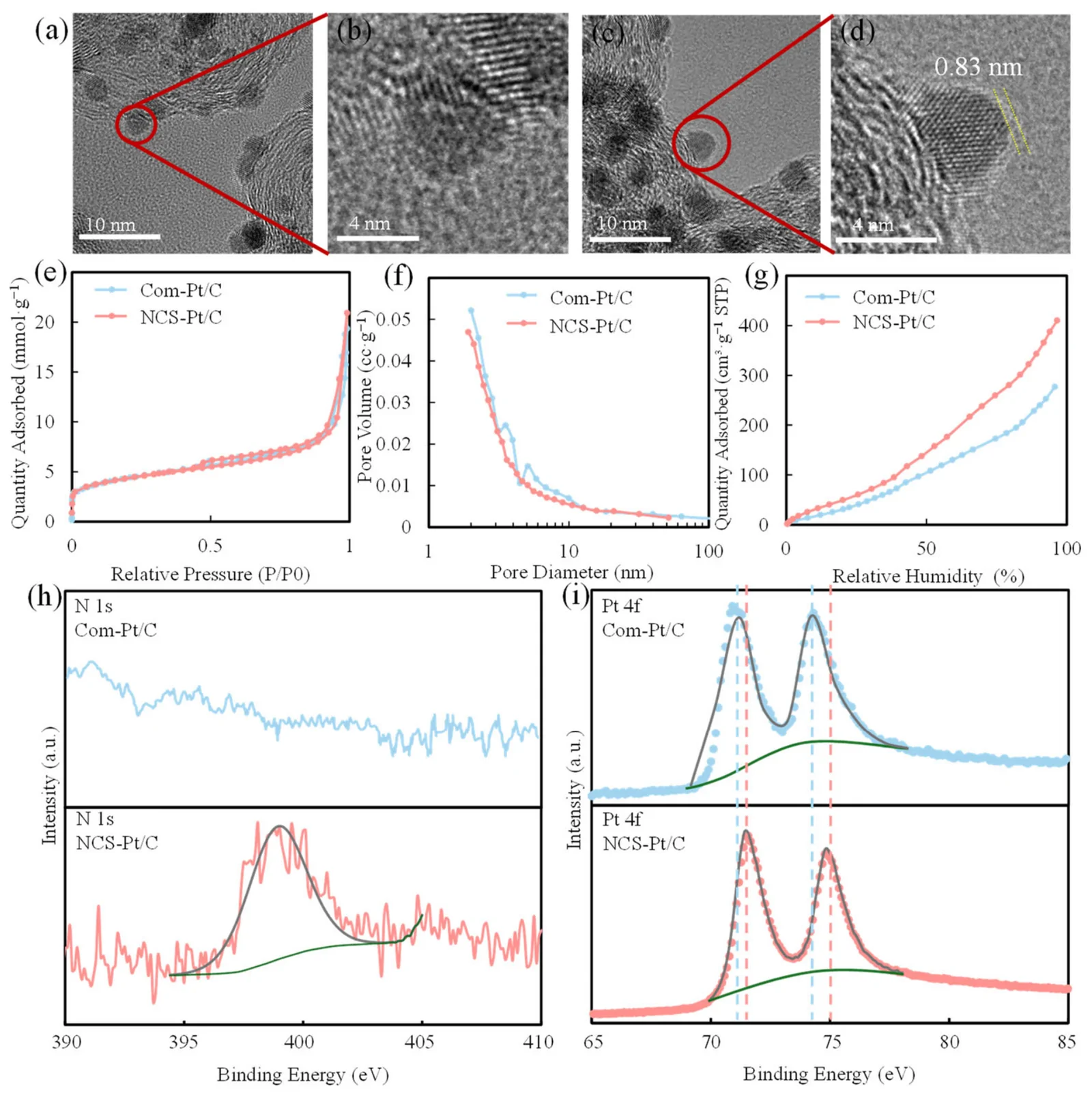
Physicochemical characterization of Com-Pt/C and NCS-Pt/C: TEM images of (**a**,**b**) Com-Pt/C and (**c**,**d**) NCS-Pt/C; (**e**) N_2_ adsorption–desorption isotherms; (**f**) BJH pore size distributions; (**g**) water vapor adsorption isotherms; and high-resolution XPS spectra of (**h**) N 1s and (**i**) Pt 4f. The yellow dashed lines in (**d**) indicate the measured thickness of the N-doped carbonaceous coating. The gray curves in (**h**) and (**i**) represent the fitted curves, while the green curves indicate the corresponding backgrounds.

The morphologies of Com-Pt/C and NCS-Pt/C were examined by TEM (Figure 2a–d). Pt nanoparticles were clearly observed on the carbon support of Com-Pt/C without a discernible surface overlayer (Figure 2a,b). After modification, an ultrathin carbonaceous layer was observed around Pt nanoparticles (in Figure 2c,d). The coating thickness was approximately 0.83 nm in the representative region shown in Figure 2d, corresponding to only a few carbon layers. Additional TEM observations from different catalyst regions consistently revealed similar ultrathin coatings, with measured thicknesses ranging from approximately 0.63 to 1.00 nm and an average value of approximately 0.81 ± 0.14 nm (Figure S1). These observations support the reproducibility of the coating structure across different regions of the modified catalyst.

Nitrogen adsorption–desorption analysis showed that the BET specific surface area decreased only modestly from 343 to 325 m^2^ g^−1^ after modification (Figure 2e). The cumulative mesopore volume in the 3–20 nm range changed from 0.22 to 0.20 cc g^−1^ (Figure 2f), indicating that the mesoporous characteristics of the parent Pt/C were largely retained after the coating treatment. In contrast, a pronounced difference was observed in water vapor adsorption. NCS-Pt/C exhibited water uptake of 72 cm^3^ g^−1^ at 30% RH and 343 cm^3^ g^−1^ at 90% RH, approximately 1.4 times that of Com-Pt/C (Figure 2g), indicating increased water affinity after surface modification.

The surface chemical composition and electronic state of the catalysts were further examined by XPS (Figure 2h,i). An N 1s signal centered near 400 eV was observed for NCS-Pt/C but was absent for Com-Pt/C, confirming the introduction of N-containing surface species during PDA-derived carbonization [37,38]. Deconvolution of the Pt 4f spectra showed that metallic Pt remained the predominant species in both Com-Pt/C and NCS-Pt/C, accounting for 62.3% and 64.3% of the total Pt 4f area, respectively. The absence of an increased oxidized-Pt fraction after modification indicates that the 450 °C treatment did not induce appreciable Pt oxidation. Meanwhile, the Pt^0^ component of NCS-Pt/C exhibited a modest positive binding-energy shift relative to Com-Pt/C, indicating a modified Pt electronic environment after formation of the N-containing carbonaceous coating [37].

### 3.2. ORR Activity of Com-Pt/C and NCS-Pt/C

**Figure 3:**
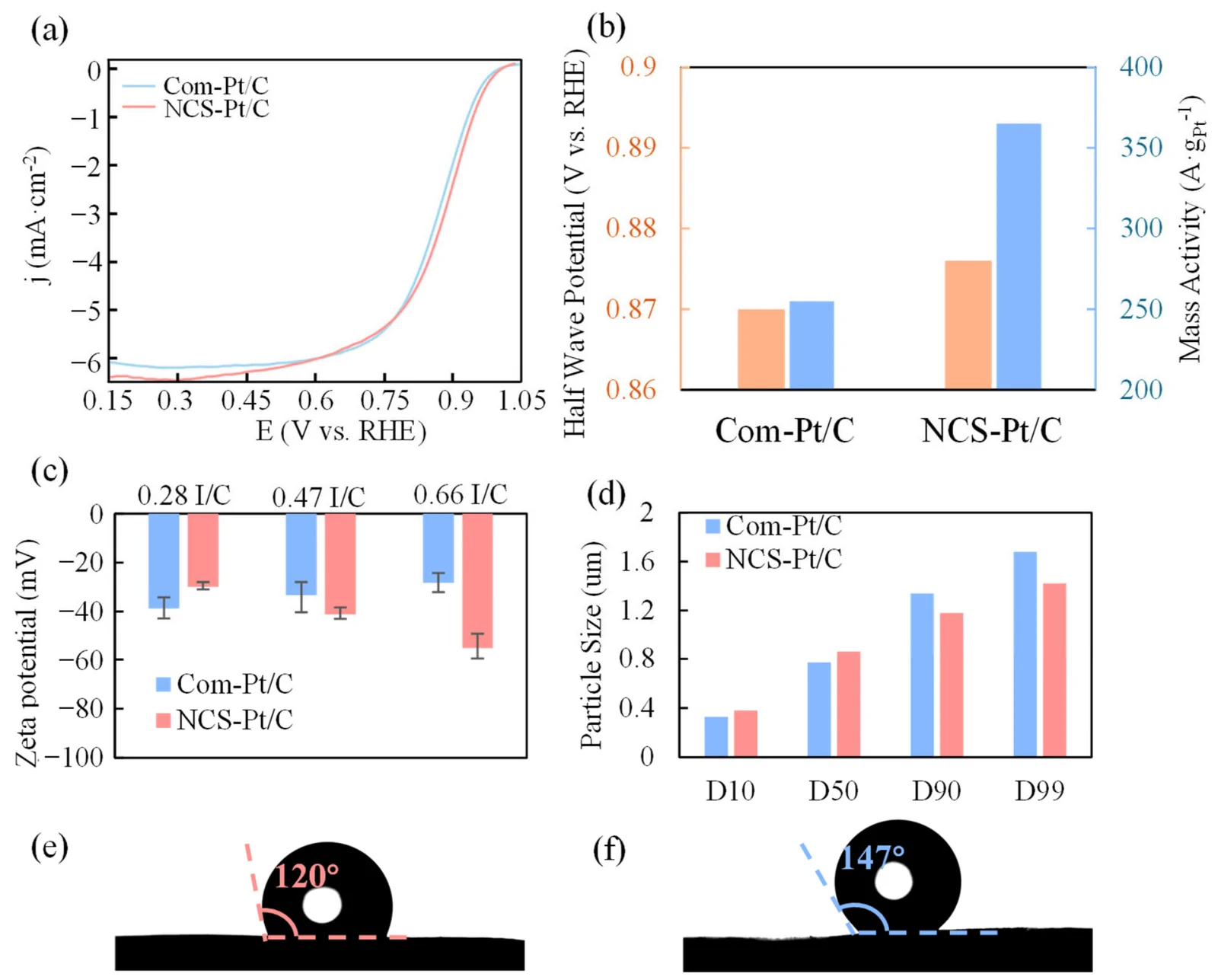
Electrochemical activity, ink properties, and catalyst-layer wettability of Com-Pt/C and NCS-Pt/C: (**a**) ORR polarization curves; (**b**) half-wave potential and mass activity at 0.90 V vs. RHE; (**c**) zeta potentials of Com-Pt/C and NCS-Pt/C catalyst inks at different I/C ratios; (**d**) particle size distributions of the catalyst agglomerates in the inks; and static water contact angles of (**e**) NCS-Pt/C CL and (**f**) Com-Pt/C CL.

NCS-Pt/C exhibited enhanced ORR activity compared with Com-Pt/C (Figure 3a,b). The half-wave potential (E_1/2_) of NCS-Pt/C reached 0.876 V vs. RHE, corresponding to a positive shift of 6 mV relative to Com-Pt/C. In addition, the mass activity at 0.90 V increased from 255 A g_Pt_^−1^ for Com-Pt/C to 362 A g_Pt_^−1^ for NCS-Pt/C, representing an improvement of approximately 42%. The corresponding RDE-derived ECSA values were 56.6 and 46.4 m^2^ g_Pt_^−1^ for Com-Pt/C and NCS-Pt/C, respectively. After normalization by ECSA, the specific ORR activity at 0.90 V increased from 4.5 A m_Pt_^−2^ for Com-Pt/C to 7.8 A m_Pt_^−2^ for NCS-Pt/C, corresponding to an increase of approximately 73%. Although NCS-Pt/C exhibited a somewhat lower ECSA, its substantially higher specific activity indicates that the improvement in mass activity cannot be attributed to differences in accessible Pt surface area alone and suggests an enhancement in intrinsic Pt surface activity. The XPS results show that the coating treatment introduces N-containing surface species and alters the electronic environment of Pt, as reflected by the positive shift in the Pt 4f binding energy. Similar PDA-derived, N-doped, carbon-coated Pt systems have been reported to exhibit Pt-N electronic interactions and a downshift of the Pt d-band center, which can modify the adsorption strength of oxygenated intermediates and promote ORR activity [37].

To evaluate whether the thermal treatment itself could account for the enhanced ORR activity, a control Pt/C catalyst was subjected to the same thermal treatment at 450 °C under N_2_ for 2 h without PDA. The thermally treated Pt/C control did not exhibit a comparable enhancement in ORR activity, including mass activity and ECSA-normalized specific activity (Figure S2), indicating that thermal treatment alone is insufficient to explain the activity improvement observed for NCS-Pt/C.

However, the present modification simultaneously introduces a carbon coating, N-containing species, thermal treatment, and changes in interfacial chemistry; therefore, the individual effects cannot be independently separated from those of the carbonaceous coating and associated interfacial changes. Although suppression of PFSA/sulfonate adsorption by ultrathin N-doped carbon layers has also been demonstrated in closely related systems [33], this effect was not directly quantified here and is, therefore, considered a possible additional contribution rather than the primary origin of the observed activity enhancement. Accordingly, the activity enhancement is discussed here as the net effect of the PDA-derived, N-containing carbon modification rather than being assigned specifically to N doping. Overall, the RDE results confirm the enhanced ORR activity of NCS-Pt/C, while the XPS results suggest that modification of the Pt electronic environment may contribute to this enhancement.

### 3.3. Catalyst Ink Dispersion and Catalyst-Layer Wettability

The behavior of the catalyst inks was examined to assess how surface modification affects catalyst–ionomer interactions prior to catalyst-layer formation (Figure 3c,d). As the I/C ratio increased from 0.28 to 0.66, the zeta potential of Com-Pt/C shifted from −39 to −28 mV, whereas NCS-Pt/C shifted from −30 to −55 mV. Thus, both catalysts remained negatively charged over the investigated I/C range, but they exhibited distinctly different responses to increasing ionomer content. The increasingly negative zeta potential of NCS-Pt/C with increasing I/C suggests that the carbon modification alters the association and interfacial configuration of the negatively charged ionomer at the catalyst-agglomerate surface. The larger magnitude of the zeta potential at higher I/C may increase electrostatic repulsion between agglomerates, which is consistent with the narrower agglomerate-size distribution observed for NCS-Pt/C (Figure 3d). The distribution span, defined as (D90 − D10)/D50, decreased from 1.31 for Com-Pt/C to 0.93 for NCS-Pt/C, with fewer large agglomerates observed for the modified catalyst [35]. Together, the zeta-potential and particle size results indicate that the carbon modification alters the dispersion behavior of the catalyst ink and the catalyst–ionomer interfacial interactions. However, these measurements reflect ensemble ink properties and do not directly resolve the spatial distribution of ionomers on the catalyst surface. Such changes in the ink state may influence the subsequent formation and transport characteristics of the catalyst layer.

The surface wettability of the catalyst layer was further evaluated by static water contact angle measurements (Figure 3e,f). The NCS-Pt/C CL exhibited an apparent contact angle of approximately 120°, compared with 147° for the Com-Pt/C CL, indicating increased surface wettability and reduced hydrophobicity after carbon modification, although both surfaces remained hydrophobic under the measurement conditions. This trend is consistent with the enhanced water vapor uptake of NCS-Pt/C (Figure 2g) and reflects the altered surface chemistry introduced by the N-containing carbon coating. It should be noted that water-vapor adsorption of the catalyst powder and contact-angle measurements of the catalyst layer provide complementary but distinct information. The former reflects the affinity of the porous catalyst toward water vapor, including vapor-accessible internal surfaces, whereas the latter reflects the apparent macroscopic wettability of the ionomer-containing catalyst-layer surface. Neither measurement alone directly quantifies the hydration state of the ionomer during fuel-cell operation. Nevertheless, the consistent increase in water affinity observed by both measurements suggests that the surface modification alters the water-interaction characteristics of the catalyst and catalyst layer. Such changes may favor local hydration under reduced-humidity conditions and are further examined through electrochemical accessibility and proton-transport measurements in the following section.

### 3.4. Humidity-Dependent Cell Performance and Transport Characteristics

**Figure 4:**
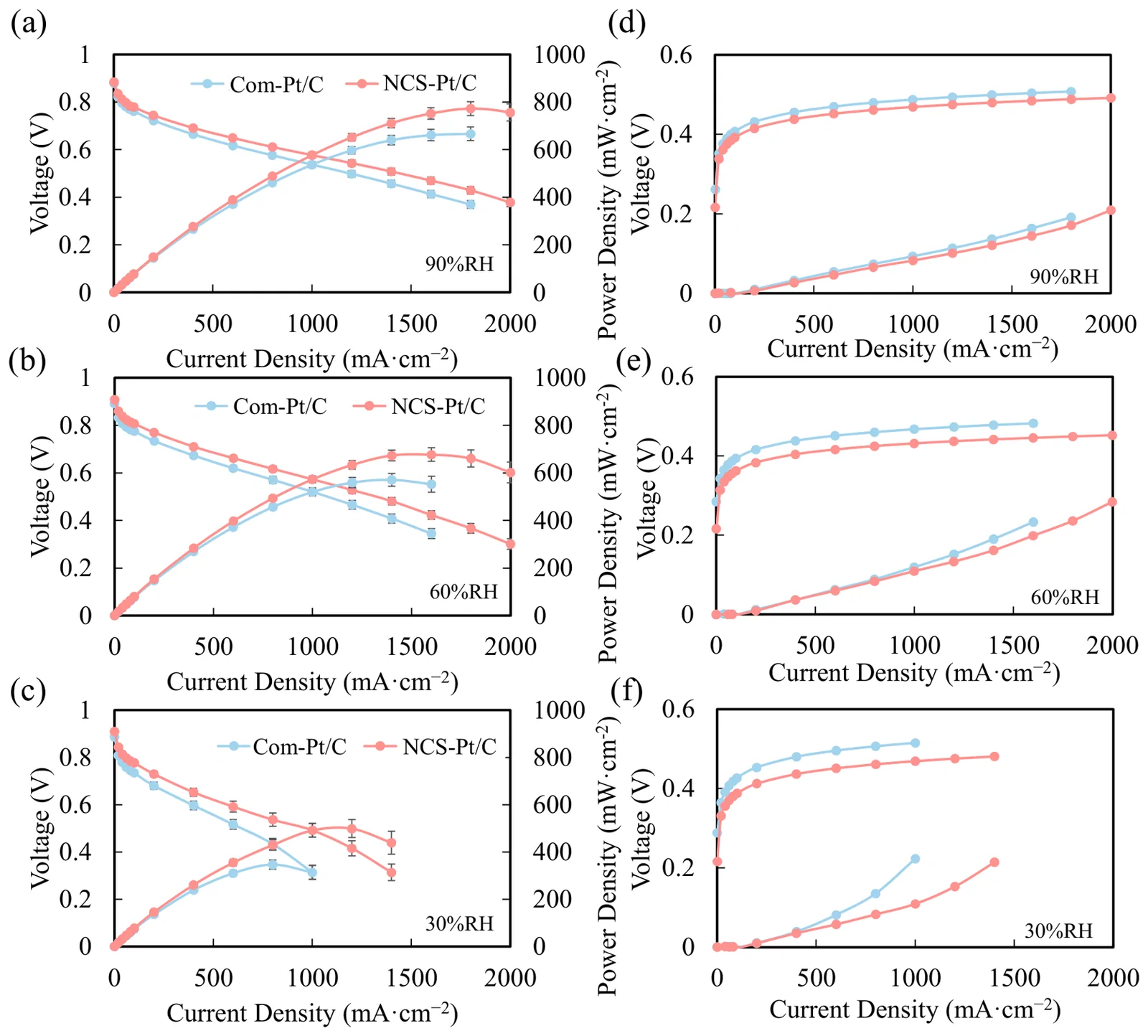
Single-cell polarization performance and voltage loss analysis of Com-Pt/C and NCS-Pt/C catalyst layers under different relative humidity conditions: polarization (data are presented as mean ± standard deviation from three repeated polarization measurements) and power density curves at (**a**) 90% RH, (**b**) 60% RH, and (**c**) 30% RH; corresponding apparent activation- and transport-related voltage losses at (**d**) 90% RH, (**e**) 60% RH, and (**f**) 30% RH.

Single-cell performance was evaluated at 30%, 60%, and 90% RH using a cathode Pt loading of 0.10 mg_Pt_ cm^−2^ (Figure 4a–c). NCS-Pt/C consistently exhibited higher cell performance than Com-Pt/C over the investigated humidity range. The peak power densities of NCS-Pt/C were 0.50, 0.67, and 0.77 W cm^−2^ at 30%, 60%, and 90% RH, respectively, compared with 0.35, 0.57, and 0.67 W cm^−2^ for Com-Pt/C. These values correspond to increases of approximately 43%, 18%, and 15%, respectively, indicating that the relative performance benefit of the carbon modification became increasingly pronounced as the RH decreased.

To further examine the origin of the performance differences, the polarization curves were analyzed by separating the apparent activation- and transport-related voltage losses according to the procedure described in Section 2.5 (Figure 4d–f). At 1000 mA cm^−2^, the apparent activation loss of NCS-Pt/C was lower than that of Com-Pt/C by 19 mV at 90% RH, with the difference increasing to 36 and 47 mV at 60% and 30% RH, respectively. The corresponding differences in the transport-related loss were 11, 10, and 114 mV. The particularly large difference observed at 30% RH indicates that the performance advantage of NCS-Pt/C under dry conditions cannot be explained solely by its modest enhancement in intrinsic ORR activity. Instead, the reduced transport-related loss is consistent with the greater water affinity of the modified catalyst layer and suggests that hydration-related transport effects contribute substantially to the low-RH performance enhancement.

**Figure 5:**
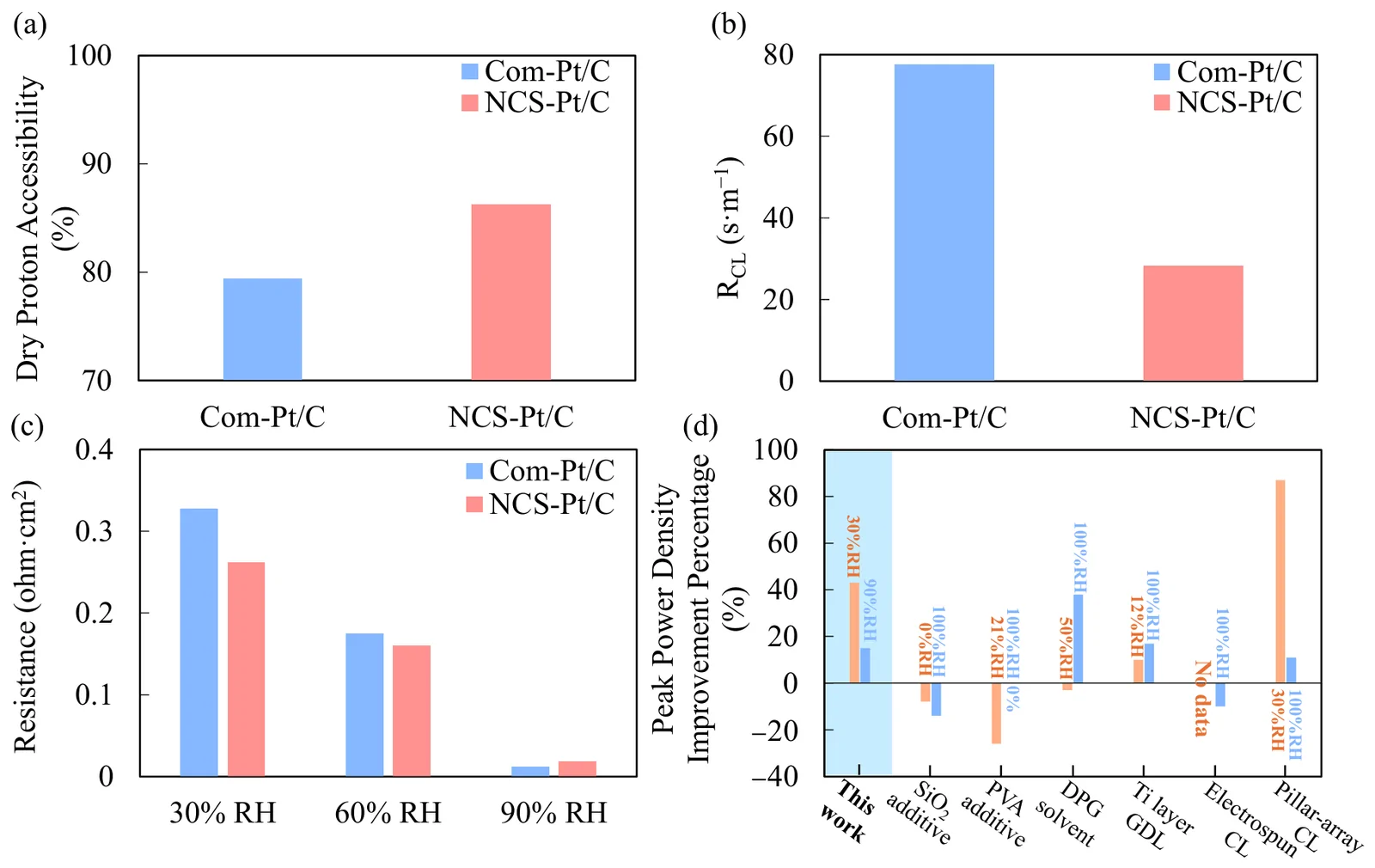
Humidity-dependent electrochemical accessibility and transport characteristics of Com-Pt/C and NCS-Pt/C catalyst layers: (**a**) dry proton accessibility, expressed as the ECSA_20%_/ECSA_100%_ ratio; (**b**) apparent catalyst-layer-associated oxygen transport resistance RCL at 90% RH; (**c**) apparent catalyst-layer proton-conduction resistance at 30%, 60%, and 90% RH; and (**d**) relative peak power density improvement of NCS-Pt/C together with selected low humidity PEMFC strategies reported in the literature (the values are shown only to provide contextual comparison; differences in electrode composition, Pt loading, membrane, operating temperature, pressure, and humidification conditions preclude direct quantitative ranking between studies). The light-blue shaded area highlights the results of the present work.

To further probe the origin of the humidity-dependent performance differences, dry proton accessibility, oxygen transport resistance, and apparent catalyst-layer proton-conduction resistance were evaluated (Figure 5a–c). Using the ECSA_20%_/ECSA_100%_ ratio as an operational indicator of dry proton accessibility, NCS-Pt/C retained a larger fraction of its ECSA upon decreasing RH. At 100% RH, the ECSA values (Figure S3) were 58.4 and 66.8 m^2^ g_Pt_^−1^ for NCS-Pt/C and Com-Pt/C, respectively, whereas at 20% RH they were 50.4 and 53.0 m^2^ g_Pt_^−1^. Accordingly, the ECSA_20%_/ECSA_100%_ ratios were approximately 86.3% for NCS-Pt/C and 79.3% for Com-Pt/C, corresponding to a difference of 7.0 percentage points. Although NCS-Pt/C exhibited a lower absolute ECSA at both humidity levels, the larger retained fraction indicates that the electrochemically accessible Pt surface was less sensitive to dehydration after carbon modification.

Oxygen transport measurements further revealed a pronounced difference between the two catalyst layers under the tested high humidity condition. At 90% RH, the apparent catalyst-layer-associated oxygen transport resistance (RCL) was 29.3 s m^−1^ for NCS-Pt/C, compared with 77.1 s m^−1^ for Com-Pt/C (Figure 5b; detailed fitting data are provided in Figure S4), corresponding to a reduction of approximately 62%. This result indicates that the carbon modification does not introduce an additional oxygen transport penalty at high RH and instead substantially lowers the apparent local transport resistance under this condition. Such behavior is consistent with modification of the catalyst–ionomer interfacial environment, although the present measurements do not distinguish the individual contributions of carbon coverage, N-containing surface species, and ionomer interaction.

A more pronounced humidity dependence was observed for apparent catalyst-layer proton-conduction resistance (Figure 5c). At 30% RH, the apparent proton-conduction resistance decreased from 0.328 ohm cm^2^ for Com-Pt/C to 0.262 ohm cm^2^ for NCS-Pt/C, corresponding to a reduction of approximately 20%. At 60% RH, the resistance decreased from 0.175 to 0.160 ohm cm^2^. In contrast, at 90% RH, NCS-Pt/C exhibited a slightly higher resistance than Com-Pt/C (0.018 vs. 0.012 ohm cm^2^). The proton transport benefit of the modification is therefore most evident under reduced humidity conditions and diminishes once the catalyst layer is sufficiently hydrated. At 90% RH, both catalyst layers are highly hydrated and exhibit very low absolute proton-conduction resistances; therefore, the higher water affinity of NCS-Pt/C does not necessarily translate into a further decrease in protonic resistance. The small difference observed at 90% RH may instead reflect differences in effective ionomer connectivity, proton-transport pathways, or experimental uncertainty. This trend is consistent with the higher water affinity of NCS-Pt/C observed from water vapor adsorption and contact angle measurements and provides a plausible explanation for the increasingly pronounced performance advantage as RH decreases.

Figure 5d and Table S1 place the present results in the context of selected strategies previously reported for improving PEMFC performance under reduced humidification. At a cathode Pt loading of 0.10 mg_Pt_ cm^−2^, NCS-Pt/C increased the peak power density by approximately 43% at 30% RH and 15% at 90% RH relative to the matched Com-Pt/C control. Because the reported studies differ in electrode composition, Pt loading, membrane properties, operating temperature, pressure, and humidification protocol, the relative improvements are used here as contextual comparisons rather than a direct performance ranking.

Taken together, these results indicate that the carbon modification is associated with reduced humidity sensitivity and concurrent changes in hydration, interfacial transport, and reaction kinetics. The stronger ECSA retention and lower apparent proton-conduction resistance under dry conditions are consistent with improved catalyst-layer hydration and proton accessibility, whereas the lower RCL measured at 90% RH demonstrates a favorable oxygen transport response under highly humidified operation. Thus, performance enhancement is more appropriately attributed to a humidity-dependent modification of the catalyst-layer hydration and transport environment, rather than a single mechanism such as uniform ionomer redistribution or suppression of sulfonate adsorption.

## 4. Conclusions

In summary, commercial Pt/C was modified with an ultrathin polydopamine-derived, N-containing carbon coating to regulate the humidity-dependent hydration and transport environment of PEMFC catalyst layers. The modification largely preserved the porous structure of the parent Pt/C while increasing water affinity and slightly enhancing intrinsic ORR activity. At a cathode Pt loading of 0.10 mg_Pt_ cm^−2^, NCS-Pt/C delivered higher single-cell performance across the investigated humidity range, with the peak power density increasing from 0.35 to 0.50 W cm^−2^ at 30% RH, corresponding to an improvement of approximately 43%. This improvement was accompanied by greater retention of the electrochemically accessible Pt surface area and lower catalyst-layer proton-conduction resistance under dry conditions, consistent with the enhanced water affinity of the modified catalyst. In addition, NCS-Pt/C exhibited substantially lower apparent catalyst-layer-associated oxygen transport resistance at 90% RH, although its proton-conduction advantage diminished under highly humidified conditions. These findings indicate that the improved low-RH performance is associated with greater retention of Pt accessibility and lower apparent catalyst-layer proton-conduction resistance under dry conditions, together with favorable oxygen transport under high-RH conditions. This work therefore provides a catalyst-level approach for improving PEMFC operation under reduced and variable humidification without requiring major changes to conventional electrode fabrication.

## Data Availability

The original contributions presented in this study are included in the article/Supplementary Material. Further inquiries can be directed to the corresponding authors.

## Supplementary Materials

The following supporting information can be downloaded at https://www.mdpi.com/article/10.3390/nano16201297/s1. Figure S1. Additional TEM images of NCS-Pt/C obtained from different catalyst regions, showing ultrathin carbonaceous coatings around Pt nanoparticles. The measured coating thicknesses are indicated in each image. Figure S2. Electrochemical characterization of the thermally treated Pt/C control (HT-Pt/C): ORR polarization curve, cyclic voltammogram, and derived electrochemical surface area (ECSA), mass activity (MA), and ECSA-normalized specific activity (SA). Figure S3. CV curves of the Com-Pt/C (blue) and NCS-Pt/C (orange) catalyst layers at 20% RH and 100% RH. Figure S4. Pressure-dependent limiting-current analysis at 90% RH: limiting-current measurements of (a) Com-Pt/C and (b) NCS-Pt/C at 100, 200, and 300 kPa absolute pressure, and (c) linear regression of total oxygen transport resistance versus absolute pressure. The intercept represents the apparent pressure-independent catalyst-layer-associated oxygen transport resistance; see Table S1. Contextual comparison of selected strategies reported for improving PEMFC performance under reduced humidity. Key electrode and operating conditions are included because differences among studies preclude direct quantitative ranking.

## Abbreviations

The following abbreviations are used in this manuscript:

AC	Alternating Current
BET	Brunauer-Emmett-Teller
BJH	Barrett-Joyner-Halenda
CL	Catalyst Layer
Com-Pt/C	Commercial Platinum-on-Carbon Catalyst
CV	Cyclic Voltammetry
DC	Direct Current
ECSA	Electrochemical Surface Area
EDS	Energy-Dispersive X-ray Spectroscopy
HT-Pt/C	Heat-Treated Platinum-on-Carbon Catalyst
HUPD	Hydrogen Underpotential Deposition
I/C	Ionomer-to-Carbon Mass Ratio
LSV	Linear Sweep Voltammetry
MA	Mass Activity
NCS-Pt/C	Nitrogen-Doped Carbon-Shell-Coated Pt/C Catalyst
ORR	Oxygen Reduction Reaction
PDA	Polydopamine
PEIS	Potentiostatic Electrochemical Impedance Spectroscopy
PEM	Proton Exchange Membrane
PEMFC	Proton Exchange Membrane Fuel Cell
PFSA	Perfluorosulfonic Acid
Pt/C	Platinum Supported on Carbon
RDE	Rotating Disk Electrode
RH	Relative Humidity
RHE	Reversible Hydrogen Electrode
SA	Specific Activity
TEM	Transmission Electron Microscopy
TGA	Thermogravimetric Analysis
XPS	X-ray Photoelectron Spectroscopy
XRF	X-ray Fluorescence Spectroscopy
