# Supplementary material for: Regulating Humidity-Dependent Catalyst-Layer Transport by an Ultrathin Carbon Interface for Low-RH PEMFCs
Source: Nanomaterials (Basel). 2026 Oct 13;16(20):1297. doi: 10.3390/nano16201297 (PMC13708550; doi:10.3390/nano16201297)
Supplement: Supplementary file 1 [file nanomaterials-16-01297-s001.zip › nanomaterials-4619306-supplementary.pdf]

Regulating Humidity-Dependent Catalyst-Layer Transport by an Ultrathin Carbon Interface for Low-RH PEMFCs

Caihan Zhu[a, b], Dewei Li[a], Tao Wu[b*], Yunan Wang[a*]

[a] *State Key Laboratory of Advanced Marine Materials, Zhejiang Key Laboratory of Advanced Fuel Cells and Electrolyzers Technology, Ningbo Institute of Materials Technology and Engineering, Chinese Academy of Sciences, Ningbo 315201, Zhejiang, China*
[b] *Department of Chemical and Environmental Engineering, University of Nottingham, Ningbo China, Ningbo 315100, China*
[*]Corresponding author.
E-mail address: wangyunan@nimte.ac.cn
E-mail address: tao.wu@nottingham.edu.cn

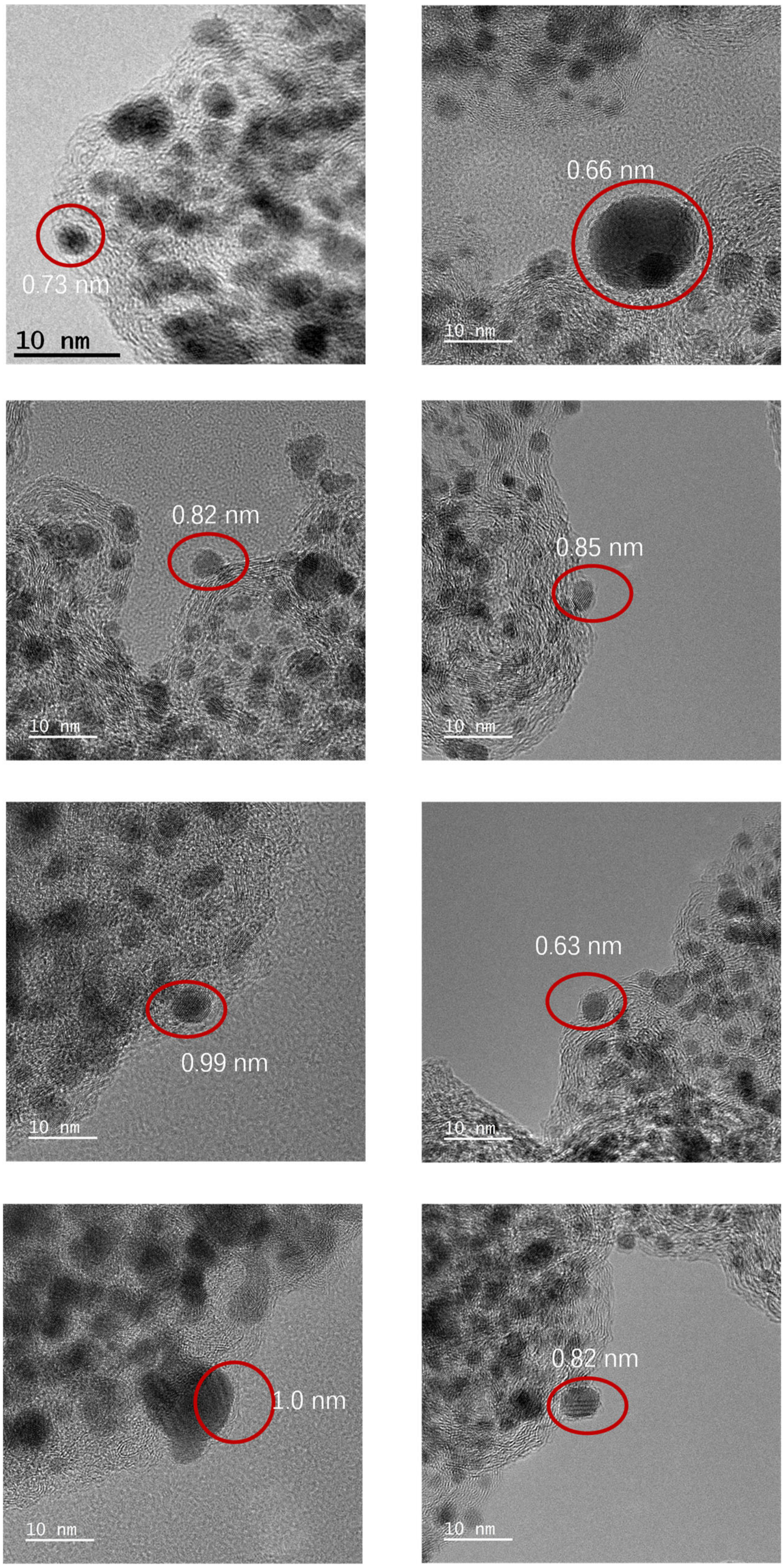

Figure S1. Additional TEM images of NCS-Pt/C obtained from different catalyst regions, showing ultrathin carbonaceous coatings around Pt nanoparticles. The measured coating thicknesses are indicated in each image.

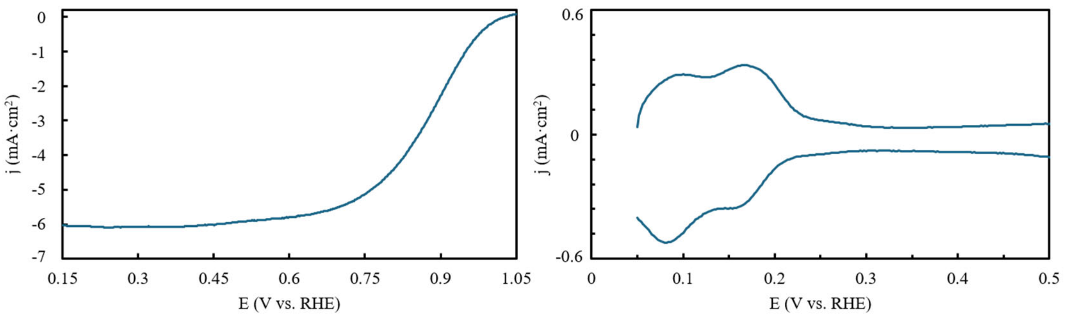

ECSA	MA	SA
47.2 m^2/g_{Pt}	293 A/g_{Pt}	6.2 A/m^2

Figure S2. Electrochemical characterization of the thermally treated Pt/C control (HT-Pt/C): ORR polarization curve, cyclic voltammogram, and derived electrochemical surface area (ECSA), mass activity (MA), and ECSA-normalized specific activity (SA).

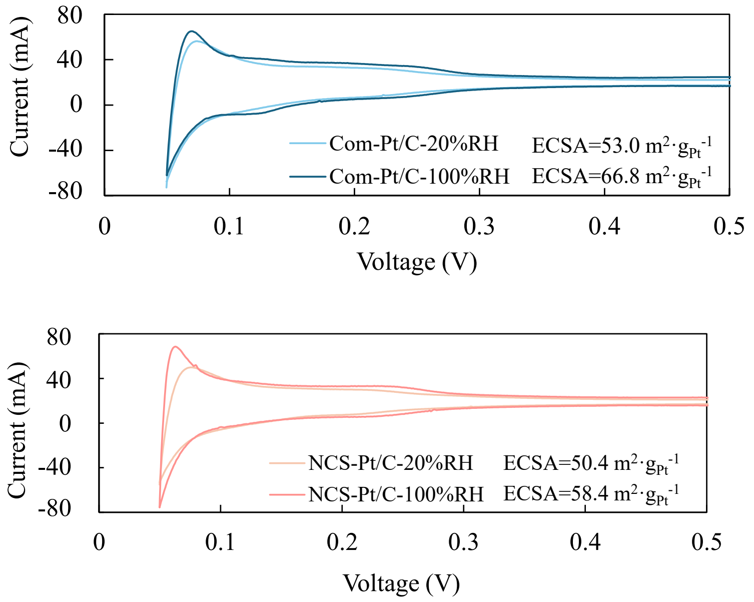

Figure S3. CV curves of the Com-Pt/C (blue) and NCS-Pt/C (orange) catalyst layers at 20% RH and 100% RH.

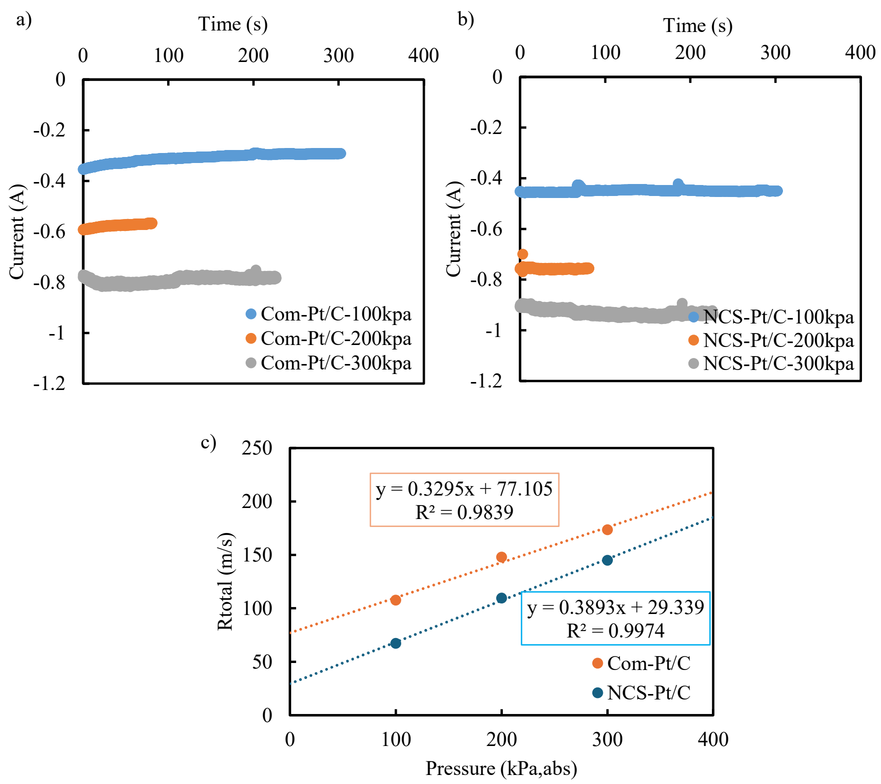

Figure S4. Pressure-dependent limiting-current analysis at 90% RH: limiting-current measurements of (a) Com-Pt/C and (b) NCS-Pt/C at 100, 200, and 300 kPa absolute pressure, and (c) linear regression of total oxygen-transport resistance versus absolute pressure. The intercept represents the apparent pressure-independent catalyst-layer-associated oxygen transport resistance.

Table S1. Contextual comparison of selected strategies reported for improving PEMFC performance under reduced humidity. Key electrode and operating conditions are included because differences among studies preclude direct quantitative ranking.

**Strategy**	**Cathode Pt loading (mgPt cm^{-2})**	**Membrane**	**Cell temperature (°C)**	**Gas pressure / backpressure**	**Fuel / oxidant**	**RH (%)**	**Potential before @ 1.0 A cm^{-2} (V)**	**Potential after @ 1.0 A cm^{-2} (V)**	**ΔV (mV)**	**Control PPD (W cm^{-2})**	**Optimized PPD (W cm^{-2})**	**PPD improvement (%)**	**Ref.**
This work	0.10	GORE-SELECT M765.08 (8.5 μm)	80	170 kPa abs	H_2 / air	30	0.31	0.49	180	0.35	0.50	43	This work
This work	0.10	GORE-SELECT M765.08 (8.5 μm)	80	170 kPa abs	H_2 / air	90	0.54	0.58	40	0.67	0.77	15	This work
γ-Al_2O_3 additive	0.60	Nafion 112	65	Ambient	H_2 / O_2	63	0.15	0.18	30	0.21	0.25	20	[22]
SiO_2 additive	0.20	NRE-212	70	/	H_2 / O_2	0	0.74	0.72	-20	0.87	0.80	-8	[26]
SiO_2 additive	0.20	NRE-212	70	/	H_2 / O_2	100	0.67	0.65	-20	1.20	1.04	-14	[26]
PVA additive	0.30	Nafion NR211	60	/	H_2 / air	21	/	/	/	0.39	0.29	-26	[25]
PVA additive	0.30	Nafion NR211	60	/	H_2 / air	100	0.58	0.58	0	0.50	0.50	0	[25]
DPG as ionomer dispersion solvent	0.25	NRE211 (25 μm)	65	No backpressure	H_2 / air	50	0.55	0.50	-50	0.60	0.58	-3	[24]
DPG as ionomer dispersion solvent	0.25	NRE211 (25 μm)	65	No backpressure	H_2 / air	100	0.56	0.60	40	0.64	0.88	38	[24]
TiO_2 layer in GDL	0.20	Nafion 212	65	30 psi backpressure	H_2 / air	12	0.60	0.63	30	0.70	0.77	10	[31]
TiO_2 layer in GDL	0.20	Nafion 212	65	30 psi backpressure	H_2 / air	100	0.62	0.64	20	0.66	0.77	17	[31]
Electrospun CL	0.10	Nafion 211	80	200 kPa abs	H_2 / air	40	/	0.56	/	/	0.62	/	[4]

Strategy	Cathode Pt loading (mgPt cm^{-2})	Membrane	Cell temperature (°C)	Gas pressure / backpressure	Fuel / oxidant	RH (%)	Potential before @ 1.0 A cm^{-2} (V)	Potential after @ 1.0 A cm^{-2} (V)	ΔV (mV)	Control PPD (W cm^{-2})	Optimized PPD (W cm^{-2})	PPD improvement (%)	Ref.
Electrospun CL	0.10	Nafion 211	80	200 kPa abs	H_2 / air	100	0.67	0.65	-20	0.72	0.65	-10	[4]
Pillar-array CL	0.40	Nafion 212 (51 μm, surface-patterned)	80	/	H_2 / air	30	/	0.42	/	0.23	0.43	87	[11]
Pillar-array CL	0.40	Nafion 212 (51 μm, surface-patterned)	80	/	H_2 / air	100	0.32	0.41	90	0.38	0.42	11	[11]

Note: "/" indicates that the corresponding parameter was not reported or was not available from the compared dataset. Ambient and no-backpressure conditions are retained as reported in the original studies.
